# Effect of hypoxic training on inflammatory and metabolic risk factors: a crossover study in healthy subjects

**DOI:** 10.1002/phy2.198

**Published:** 2014-01-13

**Authors:** Bateer Shi, Tsuneo Watanabe, Sohee Shin, Tamotsu Yabumoto, Masao Takemura, Toshio Matsuoka

**Affiliations:** 1 Department of Sports Medicine and Sports Science, Gifu University Graduate School of Medicine, 1‐1, Yanagido, Gifu, 501‐1194, Japan; 2 Department of Informative Clinical Medicine, Gifu University Graduate School of Medicine, 1‐1, Yanagido, Gifu, 501‐1194, Japan

**Keywords:** Arterial stiffness, high‐sensitivity C‐reactive protein, metabolic syndrome, pulse wave velocity

## Abstract

The purpose of this study was to investigate the influence of hypoxic physical exercise on metabolic syndrome (MS) risk markers and high‐sensitivity C‐reactive protein (CRP) and to compare its effects on preperitoneal fat, arterial stiffness, and several blood parameters related to MS to those of a control group who trained under normoxic conditions. Fourteen healthy men were examined. Participants performed treadmill exercise 3 days per week for 4 weeks, under either normobaric hypoxic or normobaric normoxic conditions, for 50 min (including a 5‐min warm‐up and 5‐min cool down) after a 30‐min rest period. Exercise was performed at a heart rate (HR) corresponding to 60% of the HR at each individual's maximum oxygen uptake. Training under the different environmental conditions was performed 4 months apart to ensure a sufficient washout period. Waist circumference, preperitoneal fat thickness, brachial‐ankle pulse wave velocity, and high‐sensitivity CRP after training were significantly lower in the hypoxic group than in the normoxic group. Our results suggest that regular short‐term hypoxic training may more effectively reduce arterial stiffness, and thus prevent arteriosclerosis, compared to training performed at a similar exercise intensity under normoxic conditions.

## Abstract (precis)

The development of atherosclerosis increases the incidence of a cardiovascular event such as heart attack or stroke. We have investigated the influence of hypoxic physical exercise on metabolic syndrome risk markers and arterial stiffness. We demonstrate that brachial‐ankle pulse wave velocity decreases in hypoxic training group.

## Introduction

Metabolic syndrome (MS) is a constellation of symptoms, including obesity, hyperglycemia, decreased high‐density lipoprotein (HDL) levels, increased triglyceride (TG) levels, and high blood pressure, and also comprises risk factors for heart disease and other health problems such as diabetes and stroke (Magliano et al. 2006). Along with these risk factors, it has been demonstrated clearly that the syndrome is common and has an increasing prevalence worldwide, which is largely associated with increasing obesity and sedentary lifestyles (Alberti et al. 2009). Moreover, increasing evidence suggests that chronic subclinical inflammation is a part of MS (Matsuo et al. 2008). Recent advances in basic science have established a fundamental role for inflammation in mediating all stages of atherosclerosis, from initiation through progression and, ultimately, to the thrombotic complications of atherosclerosis (Libby et al. 2002). The measurement of inflammatory markers such as C‐reactive protein (CRP) has been proposed as a method to assess cardiovascular risk. CRP levels, determined by using the high‐sensitivity CRP (hs‐CRP) test, can serve as an independent predictor of future cardiovascular events and add prognostic information to lipid screening (Paoletti et al. 2006).

It is well known that physical activity can reduce the risk of cardiovascular disease in individuals with established disorders such as obesity, hypertension, diabetes mellitus, and hyperlipidemia (Guo et al. 2011; Braz et al. 2012; Becker‐Grunig et al. 2013; Voulgari et al. 2013). Alternatives to traditional high‐altitude training regimens have been proposed, including the living high and training low protocol (Hahn et al. 2001), and the effects of this form of stimulus on endurance performance in athletes have been demonstrated extensively (Hendriksen and Meeuwsen 2003; Dufour et al. 2006; Roels et al. 2007). Several studies have recently reported the effect of hypoxic training on physical fitness in healthy and obese subjects (Haufe et al. 2008; Netzer et al. 2008; Vedam et al. 2009; Wiesner et al. 2010; Nishiwaki et al. 2011), including the ability of hypoxic training in reducing body weight or concentrations of blood parameters related to lipid metabolism. The potential concerning the effects of hypoxic training on lipid metabolism, Sharma (1990) reported that HDL cholesterol levels are significantly increased when living at a higher altitude. Previous research has reported that cholesterol was positively correlated with CRP and subcutaneous adipose tissue thickness of upper back (Pruller et al. 2012). Furthermore, longitudinal studies demonstrate that regular training induces a reduction in the CRP level (Mattusch et al. 2000; Okita et al. 2004). However, these studies examined the antiinflammatory effect of exercise under normoxic conditions, and not under hypoxic conditions. Thus, to our knowledge there are no published studies examining the antiinflammatory effect of regular hypoxic training using hs‐CRP. In this study, we aimed to investigate the influence of hypoxic physical exercise on MS risk markers and hs‐CRP levels and to compare its effects on preperitoneal fat, arterial stiffness, and several blood parameters related to MS to those of a control group who were trained under normoxic conditions.

## Methods

### Subjects

Seventeen participants were recruited, of whom 14 completed the study. Three subjects dropped out of the study: two individuals because of knee injury (not incurred during the experiment) and one because of the lack of motivation. Fourteen healthy men (age: 27.4 ± 2.6 years, height: 174.4 ± 1.4 cm, weight: 70.3 ± 3.0 kg, body mass index [BMI]: 23.1 ± 1.0 kg m^−2^), with no history of cardiopulmonary disease and no pathogenic conditions affecting the musculoskeletal or neuromuscular systems, volunteered to participate in this study. Furthermore, women were not included in this study because some blood parameters have a sex difference. Prior to the study, all subjects underwent a medical examination, including echocardiography and electrocardiography at rest. Briefly, M‐mode and two‐dimensional echocardiography were performed in order to except cardiovascular disease as ischemic heart disease. Echocardiography and electrocardiography were performed using a portable real‐time apparatus (LOGIQ *e*; GE Healthcare Co., Tokyo, Japan), and a FDX‐4521 (FUKUDA DENSHI Co., Ltd., Tokyo, Japan). In all 14 subjects, spiroergometry was performed to determine maximum oxygen uptake (*V*O_2max_) during exercise and to determine a study training intensity, which was set at a heart rate (HR) corresponding to 60% of that at *V*O_2max_. Participants were asked to continue with their normal prestudy diet. All subjects provided informed consent as required by the university institutional review board, which approved the study.

### Protocol

This study had a same‐subjects repeated measures with crossover study design. Subjects were randomly divided into two groups that were matched for initial physical exercise performance. Seven were assigned to the hypoxic group and the other seven were assigned to the control group (normoxic group). Training intervention was for 4 weeks, and the subjects were asked to switch groups 4 months after they completed the first intervention.

Experiments were conducted in a controlled environment with the temperature maintained at 22°C. Training under the different environmental conditions was performed 4 months apart to ensure a sufficient washout period. Participants performed treadmill exercise 3 days per week for 4 weeks, under either normobaric hypoxic (15.4% O_2_, equivalent to an altitude of 2500 m) or normobaric normoxic (20.9% O_2_, equivalent to sea level) conditions, for 50 min (including a 5‐min warm‐up and 5‐min cool down) after a 30‐min rest period. Before and after rest periods were performed at each environmental condition. Exercise was performed at a HR corresponding to 60% of the HR at each individual's *V*O_2max_. All subjects were required to rest for 30 min after exercise. During the exercise session, HR was monitored to ensure that exercise intensity never exceeded 60% of the maximum HR. Subjects were asked to report any symptoms of acute altitude sickness (e.g., headache, nausea, atypical weakness in the legs), and percutaneous oxygen saturation (SpO_2_) was also monitored for safety. The SpO_2_ was measured using a PULSOX‐M24 pulse oximeter (TEIJIN PHARMA LIMITED, Tokyo, Japan). Laboratory parameters were determined at the beginning and end of the 4‐week trial; height, body weight, and body fat were measured via a regular medical scale. Blood pressure was measured using a digital blood pressure meter (HEM‐7051‐HP; OMRON HEALTHCARE Co., Ltd., Kyoto, Japan).

### Data collection

#### Anthropometric measurement

Anthropometric measurements were performed by trained staff with the participants barefoot and in their underwear. Waist circumference was measured to the nearest 0.1 cm using a vinyl tape, at the narrowest circumference between the lowest ribs and iliac crests in the standing position. The height was measured using an analog‐type stadiometer to the nearest 0.1 cm. Body weight and body fat were measured to the nearest 0.1 kg using a BC‐118D body composition analyzer (TANITA Co., Tokyo, Japan). Percentage body fat was evaluated by means of electrical impedance analysis. BMI was calculated as (body weight [kg]/height [m^2^]).

#### Blood sampling

Subjects were asked to fast from 11:00 pm, the evening before visiting the laboratory. A fasting blood sample was taken at noon on the day of the experiment from an antecubital vein, with the patient in sitting position. Study parameters included total cholesterol (TC), HDL cholesterol, low‐density lipoprotein (LDL) cholesterol, TG, fasting glucose, fasting insulin, leptin, adiponectin, hs‐CRP, and Interleukin (IL)‐6. TC was analyzed using an enzymatic method, HDL and LDL were analyzed using direct methods, and TG was analyzed using elimination of free glycerol interference with a colorimetric enzymatic assay using a JCA‐BM6070 automated biochemical analyzer (JEOL Ltd., Tokyo, Japan). Insulin was measured using a chromogenic assay with a LUMIPULSE G1200 autoimmunoanalyzer (Fujirebio Inc., Tokyo, Japan). Glucose was measured via enzyme assay using a GA09 (A&T Co., Yokohama, Japan). Leptin was analyzed with an ARC‐950 analyzer (Hitachi Aloka Medical Ltd., Tokyo, Japan) using a double‐antibody radioimmunoassay. Adiponectin was analyzed with a JCA‐BM12 automatic analyzer (JEOL Ltd.) using latex agglutination turbidimetry. Hs‐CRP was analyzed using nephelometry with the BN™ II System (Siemens Healthcare Diagnostics Co., Ltd., Tokyo, Japan), whereas IL‐6 was analyzed using enzyme‐linked immunosorbent assay (ELISA) (eBioscience Inc., San Diego, CA). Intraassay coefficients of variation were below 3.0% for TC, TG, and hs‐CRP; below 5.0% for HDL, LDL, and glucose; and below 10% for insulin, leptin, adiponectin, and IL‐6.

#### Measurement of *V*O_2max_

Oxygen consumption was measured using a *V*_max_ 29 analyzer (CareFusion Co., Yorba Linda, CA). *V*O_2max_ was measured using the modified Astrand protocol (Pollock et al. 1984), in which participants ran at 70% HRmax for 3 min and the gradient was then increased by 2% every 2 min until subject exhaustion. Although the use of this protocol and a constant speed is associated with a slight risk of falling while running, *V*O_2max_ can be measured accurately within a short time. *V*O_2_ was recorded continuously using a *breath‐by‐breath* method and HR was measured simultaneously. The values of *V*O_2max_ were considered acceptable if the subjects met at least two of following criteria: an oxygen uptake plateau, highest respiratory exchange ratio >1.1, peak HR within five beats of the age‐predicted maximum (220 – age), and a rating of perceived exertion of >19 on the Borg scale.

#### Measurement of arterial stiffness

Ankle‐brachial pulse wave velocity (PWV) and ankle‐brachial index (ABI), representing arterial stiffness, were measured noninvasively with subjects in the supine position using a form‐I automated PWV/ABI analyzer (Colin Co. Ltd., Komaki, Japan) attached to the four limbs (Yamashina et al. 2002). PWV is generally assessed by measuring the time the pulse wave takes to travel a given distance along the blood vessel and can provide an objective index of atherosclerosis (Lehmann et al. 1998).

#### Measurement of preperitoneal fat thickness

**Figure 1.**
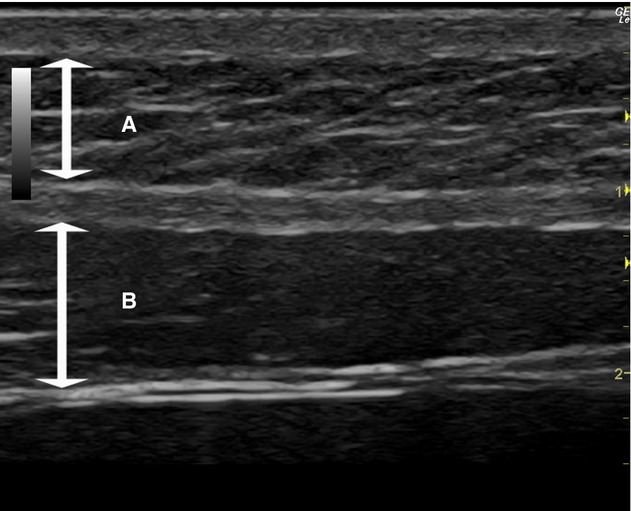
Ultrasonogram of the upper abdominal wall on the xiphoumbilical line. (A) Subcutaneous fat thickness, (B) preperitoneal fat thickness.

Preperitoneal fat thickness (PFT) was evaluated sonographically using a 6.0–14.0 MHz linear array probe (portable real‐time apparatus: LOGIQ *e*; GE Healthcare Co.). Ultrasound examination of abdomen was performed by a board certified sonographer who was blinded to the group assignment. Both preperitoneal and subcutaneous fat thicknesses (SFT) were measured according to the criteria of Suzuki et al. (1993). Longitudinal scanning was done from the xiphoid process to the umbilicus along the linea alba of subject in a supine position. The probe was maintained to make contact as lightly as possible so as not to compress the fat layer. The PFT and the SFT were measured (Fig. 1). We conducted a preliminary examination in five healthy participants in order to confirm the intraobserver reliability using calculations of intraclass correlation coefficients (ICC; one‐way analysis of variance). PFT and SFT were repeated five times for each subject, intraobserver reproducibility was high for both parameters (PFT: ICC = 0.949; SFT: ICC = 0.994). An ICC value of >0.9 was considered very good.

### Statistical analysis

Results are presented as mean ± standard error (SE), and statistical significance was indicated by *P *<**0.05. The adequacy of the sample size was justified with power analysis calculations performed using G*power 3.1 (G*Power; Erdfelder et al. 1996). A two‐way factorial analysis of variance (ANOVA) test was used to determine differences between several parameters (2 × 2; normoxia/hypoxia × before/after training). Tukey's honestly significant difference test was applied to assess differences among multiple comparisons when ANOVA indicated a significant difference for a particular factor. When data were not normally distributed, nonparametric statistics such as Wilcoxon's test was applied. The statistical program used for the calculations was IBM SPSS Statistics 19.0 (IBM SPSS, Chicago, IL).

## Results

Demographic and physiological baseline characteristics for both training groups at the start of the study are given in Table 1. Before training, no significant differences in any of the values were identified between the groups. Normal distribution was first assessed. The ANOVA test was used to determine differences between several parameters whose data were assumed to have normal distribution. If data were not assumed to have normal distribution, a nonparametric test was applied. Measurements for several parameters according to group as well as before and after training are shown in Table 2.

**Table 1.** Characteristics at the start of the study of the 14 participants who completed the study.

	Normoxic group	Hypoxic group	*P*‐value
Men, *n*	14	14	N.A
Height, cm	174.4 ± 1.5	174.4 ± 1.5	N.A
Body weight, kg	70.3 ± 3.1	70.2 ± 3.1	0.985
BMI, kg m^−2^	23.1 ± 0.9	23.1 ± 1.1	0.996
Body fat, %	20.3 ± 1.9	19.5 ± 2.0	0.772
*V*O_2max_, mL·min^−1^·kg^−1^	54.1 ± 2.5	51.7 ± 2.4	0.482

Values are means ± SE. BMI, body mass index; *V*O_2max_, maximum oxygen uptake; N.A, not applicable.

**Table 2.** Physical and several laboratory parameters of the subjects, before and after training.

Parameter	Group	Training		Two‐way ANOVA (*F*‐value)			Post hoc test (Tukey's HSD)
	(*n*)	Before	After	Group	Training	Interaction	
Weight, kg	Normoxia (*n* = 14)	70.3 ± 3.1	69.9 ± 3.0	0.23	3.89	1.03	NS
	Hypoxia (*n* = 14)	70.2 ± 3.4	69.3 ± 3.2				
Percentage Body fat, %	Normoxia (*n* = 14)	20.3 ± 1.9	20.6 ± 2.0	6.49	0.35	3.13	After training: hypoxia < normoxia
	Hypoxia (*n* = 14)	19.5 ± 2.0	18.8 ± 2.0	(*P* = 0.024)			
Waist Circumference, cm	Normoxia (*n* = 14)	81.8 ± 3.3	80.9 ± 2.9	0.03	6.21	1.73	Hypoxia group: after < before training
	Hypoxia (*n* = 14)	82.4 ± 3.5	80.6 ± 3.3	(*P* = 0.024)			
BMI, kg m^−2^	Normoxia (*n* = 14)	23.1 ± 1.0	22.9 ± 1.0	0.19	5.02	1.52	NS
	Hypoxia (*n* = 14)	23.1 ± 1.1	22.8 ± 1.0		(*P* = 0.043)		
PWV, cm sec^−1^	Normoxia (*n* = 14)	1118.2 ± 47.2	1129.6 ± 47.0	3.17	0.78	4.84	After training: hypoxia < normoxia
	Hypoxia (*n* = 14)	1109.1 ± 45.8	1066.9 ± 40.7			(*P* = 0.046)	
SFT, mm	Normoxia (*n* = 14)	7.0 ± 1.6	6.8 ± 1.6	4.24	6.70	0.13	After training: hypoxia < normoxia
	Hypoxia (*n* = 14)	6.0 ± 1.3	5.7 ± 1.3		(*P* = 0.023)		
PFT, mm	Normoxia (*n* = 14)	11.1 ± 1.5	10.1 ± 1.4	0.55	16.89	0.24	Hypoxia group: after < before training
	Hypoxia (*n* = 14)	10.9 ± 1.5	9.6 ± 1.5		(*P* = 0.001)		
TC, mg dL^−1^	Normoxia (*n* = 14)	181.8 ± 7.6	184.9 ± 7.8	3.06	0.54	0.10	NS
	Hypoxia (*n* = 14)	174.3 ± 6.6	175.7 ± 6.5				
HDL, mg dL^−1^	Normoxia (*n* = 14)	54.7 ± 3.1	59.2 ± 3.3	6.57	10.28	0.59	NS
	Hypoxia (*n* = 14)	51.7 ± 3.2	54.4 ± 2.6	(*P* = 0.025)	(*P* = 0.008)		
LDL, mg dL^−1^	Normoxia (*n* = 14)	107.1 ± 7.6	108.4 ± 8.1	0.79	1.40	0.34	NS
	Hypoxia (*n* = 14)	102.4 ± 6.2	106.4 ± 7.4				
Adiponectin, *u*g mL^−1^	Normoxia (*n* = 14)	7.82 ± 0.84	8.02 ± 0.98	0.09	0.03	0.53	NS
	Hypoxia (*n* = 14)	7.86 ± 0.78	7.74 ± 0.96				

Values are means ± SE. BMI, body mass index; PWV, brachial‐ankle pulse wave velocity; PFT, preperitoneal fat thickness; SFT, subcutaneous fat thickness; TC, total cholesterol; HDL, high‐density lipoprotein cholesterol; LDL, low‐density lipoprotein cholesterol; NS, not significant.

### Anthropometric data

No significant main effects or interactions were observed for body weight. Despite the absence of any significant main effects among the groups, a significant main effect according to training conditions (before and after) was observed for BMI [*F*(1, 13) = 5.02, *P = *0.043]. Moreover, despite the absence of any significant main effects between training conditions, a significant main effect according to group was observed for percentage body fat [*F*(1, 13) = 6.49, *P = *0.024] and waist circumference [*F*(1, 13) = 0.03, *P = *0.024]. Subsequently, percentage body fat after training was significantly lower in the hypoxic group than in the normoxic group. Moreover, waist circumference in the hypoxic group was significantly lesser after training than before training.

### Metabolic risk markers

Significant main effects according to groups and training conditions were observed for HDL [*F*(1, 13) = 6.57, *P = *0.025; *F*(1, 13) = 10.28, *P = *0.008], whereas no significant main effects or interactions were observed for TC and LDL. The nonparametric statistics results are shown in Table 3. The blood pressure, glucose, insulin, and TG values were not normally distributed. Moreover, no significant differences were observed for any metabolic risk parameters between before and after training in both the normoxic and hypoxic groups.

**Table 3.** Comparison between percentage change in several parameters before and after training

Parameters	Group	Training		Percentage change	Wilcoxon's test
		Before	After	(%)	(*P*‐Value)
SBP, mmHg	Normoxia	124.1 ± 1.9	121.8 ± 2.3	−1.8 ± 1.8	0.363
	Hypoxia	119.1 ± 3.1	120.7 ± 2.6	1.7 ± 2.0	
DBP, mmHg	Normoxia	72.1 ± 1.8	71.4 ± 2.0	−1.0 ± 1.2	0.972
	Hypoxia	70.9 ± 3.2	69.5 ± 2.0	−0.4 ± 3.3	
Glucose, mg dL^−1^	Normoxia	90.4 ± 2.5	90.4 ± 2.5	0.2 ± 1.9	0.140
	Hypoxia	92.4 ± 3.2	96.6 ± 3.0	5.5 ± 3.5	
Insulin, *u*U mL^−1^	Normoxia	5.3 ± 0.7	3.9 ± 0.5	−15.9 ± 9.9	0.638
	Hypoxia	10.3 ± 3.5	6.0 ± 1.2	−3.0 ± 19.8	
TG, mg dL^−1^	Normoxia	105.6 ± 15.8	88.8 ± 12.8	−2.2 ± 11.2	0.245
	Hypoxia	122.7 ± 35.1	81.8 ± 16.5	−18.7 ± 9.1	
Leptin, ng mL^−1^	Normoxia	3.84 ± 1.03	2.82 ± 0.61	−12.4 ± 12.6	0.638
	Hypoxia	4.30 ± 1.28	3.32 ± 1.10	−19.2 ± 8.8	
hs‐CRP, ng mL^−1^	Normoxia	448.6 ± 117.3	512.9 ± 254.1	32.8 ± 44.1	0.013
	Hypoxia	664.3 ± 241.5	207.1 ± 51.6	−41.4 ± 13.6	
IL‐6, pg mL^−1^	Normoxia	1.9 ± 1.3	8.3 ± 6.4	339.7 ± 295.3	0.091
	Hypoxia	18.2 ± 16.8	13.1 ± 12.3	−28.9 ± 24.5	

Values are means ± SE. SBP, systolic blood pressure; DBP, diastolic blood pressure; TG, triglycerides; hs‐CRP, high‐sensitive C‐reactive protein; IL‐6, Interleukin‐6.

### Arterial stiffness

**Figure 2.**
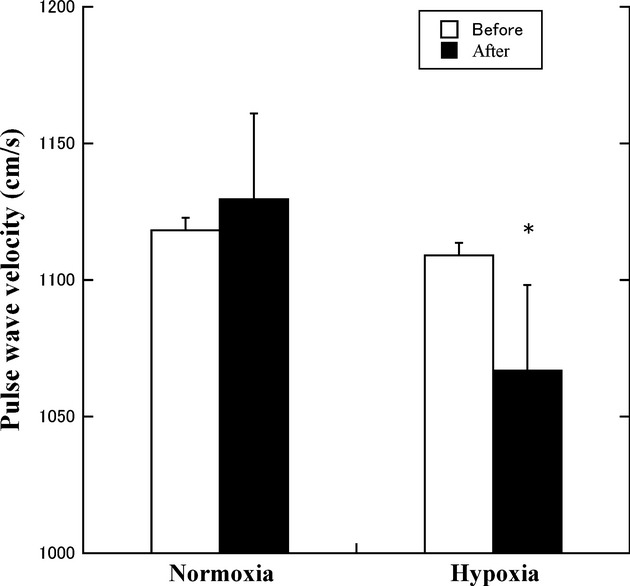
Change in the pulse wave velocity values in the normoxic and hypoxic group. *P <**0.05 versus before training in the hypoxic group.

Significant interactions were observed for PWV [*F*(1, 13) = 4.84, *P = *0.046]. Subsequently, PWV after training was significantly lower in the hypoxic group than in the normoxic group (*P *=**0.014) (Fig. 2).

### Preperitoneal fat thickness

**Figure 3.**
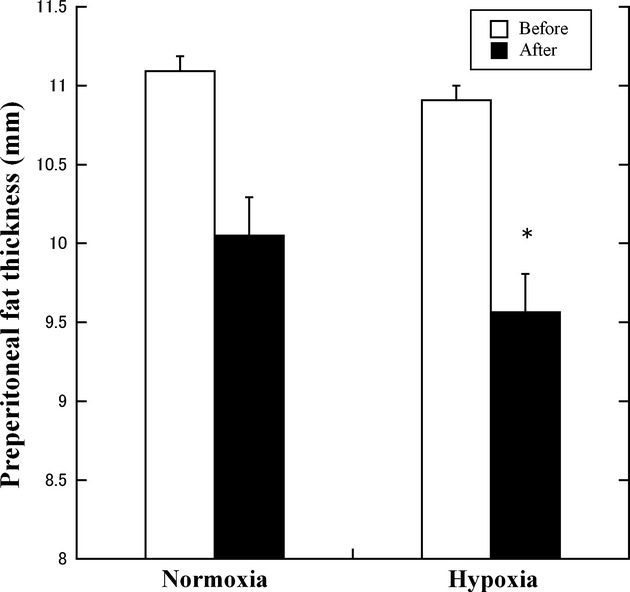
Change in the preperitoneal fat thickness values in the normoxic and hypoxic group. *P <**0.05 versus before training in the hypoxic group.

Despite no significant main effects among the groups, a significant main effect for before–after training was observed for SFT [*F*(1, 13) = 6.70, *P = *0.023] and PFT [*F*(1, 13) = 16.89, *P = *0.001]. Subsequently, the SFT after training was significantly lower in hypoxic group than in normoxic group (*P *<**0.001). The PFT in hypoxic group was significantly lower after training than in before training (*P *=**0.037) (Fig. 3).

### Inflammatory markers (including adipocytokines)

**Figure 4.**
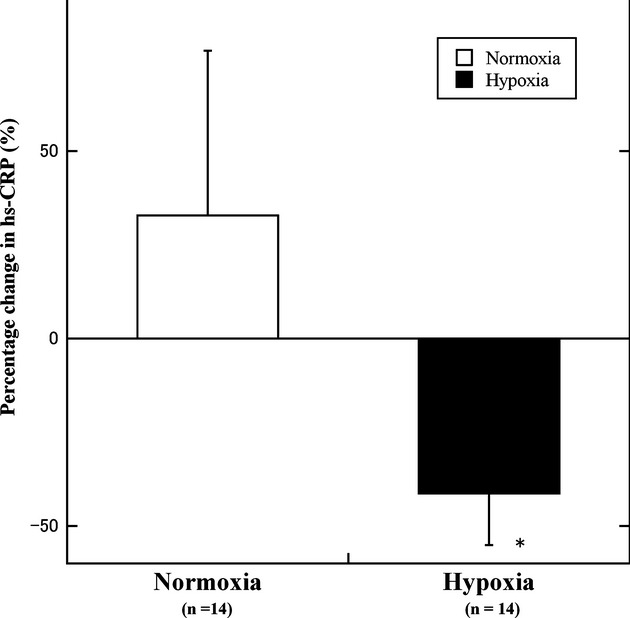
Change in the hs‐CRP levels in the normoxic and hypoxic group. Hs‐CRP, high‐sensitivity C‐reactive protein. *P <**0.05 versus the normoxic group.

No significant main effects or interactions were observed for adiponectin. The leptin, hs‐CRP, and IL‐6 values were not normally distributed. Hs‐CRP levels were significantly lower in the hypoxic group compared to the normoxic group (*P = *0.013) (Fig. 4). The leptin and IL‐6 values decreased to a greater extent in the hypoxic group than in the normoxic group, although the difference was not significant.

## Discussion

This study investigates the influence of short‐term hypoxic physical exercise on metabolic risk markers, inflammatory markers such as hs‐CRP, and arterial stiffness in healthy subjects, and to our knowledge, this is the first such study to employ a crossover study design under controlled laboratory conditions. The study demonstrated the effects on MS‐related markers such as decreased waist circumference and PFT, and a significant decrease in PWV and hs‐CRP levels in subjects who exercised under hypoxic conditions. Our results suggest that short‐term hypoxic exercise is associated with the prevention of not only accumulation of abdominal visceral fat but also arterial stiffness.

The development of atherosclerosis increases the incidence of a cardiovascular event such as heart attack or stroke. A study by Hirai et al. (1989) showed strong associations between aortic stiffness and the degree of coronary artery disease, and similar associations have been reported between PWV and independently assessed cardiovascular risk scores (Lehmann et al. 1998). PWV is an established index of atherosclerosis and also a marker for atherosclerosis (van Popele et al. 2001). In contrast, endothelial dysfunction is involved in every stage of the progression of atherosclerosis (Grundy et al. 1999) and is noninvasively measurement by flow‐mediated dilation (FMD) of brachial artery (Corretti et al. 2002). Recently, Liu et al. (2006) reported that increased brachial‐ankle PWV is related impaired endothelial dysfunction in patients with coronary artery disease, it has been suggested that PWV can be used a noninvasive surrogate index in clinical evaluation of endothelial function. Nishiwaki et al. (2011) demonstrated a significant increase in the percentage of FMD and a significant reduction in PWV in the hypoxic group after training in postmenopausal women. Their results indicate that hypoxic training may induce vascular functional adaptation. Katayama et al. (2013) also investigated the effect of acute hypoxic exercise on FMD and reported that % FMD was higher in the hypoxic exercise group. They suggested that aerobic exercise under hypoxic conditions has a significant impact on endothelial‐mediated vasodilatation. Our results also showed that PWV was significantly decreased in the post–hypoxic training group compared to the normoxic training group, which are in agreement with those of previous reports.

The inflammatory component of atherogenesis has been increasingly recognized over the last decade. It is well known that MS leads to cardiovascular disease (Aballay et al. 2013). Central obesity and insulin resistance are believed to represent common underlying factors of the syndrome, which features a chronic low‐grade inflammatory state (Paoletti et al. 2006). Low‐grade chronic inflammation is reflected by increased CRP concentration and increased levels of certain cytokines (Ross 1999). The presence of the antiinflammatory effect of exercise has been indicated by changes in the levels of cytokine such as tumor necrosis factor alpha and IL‐6, as noted in a review article by Petersen and Pedersen (2005). Hammett et al. (2004) reported that 6 months of exercise training had no effect on CRP, whereas Okita et al. (2004) showed that CRP levels were significantly decreased by 34.9% after a 2‐month aerobic exercise program. Thus, there are inconsistencies among current studies on the effect of normoxic training on CRP reduction. Furthermore, few studies have examined the effect of hypoxic exercise training on CRP. At the molecular level, hypoxia results in an increased level of the stress protein endothelial heme oxygenase‐1 (HO‐1) (Motterlini et al. 2000). HO‐1 is a rate‐limiting enzyme in the catabolism of heme, which plays a critical role in the prevention of vascular inflammation (Abraham and Kappas 2008). Indeed, HO‐1 may be involved in the most critical cytoprotective mechanisms that are activated during times of cellular stress such as inflammation, ischemia, and hypoxia (Choi and Alam 1996), and the opposite effect of HO‐1 in atherogenesis has clearly been indicated in previous studies (Araujo et al. 2012). Although this study demonstrated a significant reduction in the hs‐CRP levels in the hypoxic group, hs‐CRP levels did not change significantly in the normoxic group during the study period. In addition, IL‐6 levels tended to decrease to a greater extent in the hypoxic group, although not to a significant degree, than in the normoxic group. Moy et al. (2013) reported a significant linear trend of increasing daily step count by quartiles and decreasing CRP and IL‐6 levels. Thus, the authors hypothesize that hypoxic aerobic exercise has a stronger effect on antiinflammation due to environmental effects compared to normoxic aerobic exercise.

The values of two MS risk markers, percentage of body fat and waist circumference, were significantly reduced after training compared with the values before training in the hypoxic group. In addition, PFT was significantly reduced after training compared with the value before training in the hypoxic training group. It is generally known that intraabdominal visceral fat accumulation plays a major role in the development of MS or cardiovascular disease. Although computed tomography (CT) scanning is the standard method for determining visceral fat volume in the abdomen, it is not widely available, involves the use of ionizing radiation, and is considered to be a somewhat costly examination. However, ultrasonographic method is highly useful because of the low cost, lack of radiation exposure, and high correlation with CT findings (Leite et al. 2002). In this study, compared with normoxic training, PFT, measured ultrasonographically, and waist circumference in the hypoxic group were significantly lower after training than that before training. Although the difference was not significant, the levels of leptin – a hormone secreted by adipose tissue – decreased to a greater extent in the hypoxic group than in the normoxic group. Leptin concentrations have been reported to be associated with BMI and body fat (Ryan and Elahi 1996). Furthermore, Kobayashi et al. (2004) showed the relationship between intraabdominal visceral fat mass by CT and serum leptin. Thus, our findings may reveal that hypoxic training is useful in the prevention of MS. However, reductions in lipid levels or glucose metabolism parameters were not observed after training in either the normoxic or hypoxic training group. Several authors have investigated whether hypoxic training can reduce MS‐related markers (Haufe et al. 2008; Netzer et al. 2008; Wiesner et al. 2010). Netzer et al. (2008) found no significant effect on either lipid or glycogenated hemoglobin levels after normobaric hypoxic training using low‐intensity physical exercise (60% *V*O_2max_), which was consistent with our results. Wiesner et al. (2010) also investigated the hypoxic training effect on MS risk markers in obese subjects. Their results show that hypoxic exercise had no significant influence on blood pressure or LDL cholesterol levels. Our findings are also in agreement with previous studies by Wiesner et al. (2010) that examined the effect of hypoxic training on MS risk markers. The underlying mechanism through which hypoxic training influences the visceral fat volume remains unclear. Further studies with larger numbers of participants are needed for confirmation.

Our study has several limitations. First, the subjects were healthy young men; thus, the study did not evaluate the reduction in MS risk markers due to regular hypoxic training in patients with metabolic disorders. In addition, women were not included in this study. In general, gender differences in changes to arterial stiffness are huge because estrogen has potent vasodilatory and antiatherosclerotic effects in vascular tissue; thus, the study did not evaluate the effects of sex differences. Percentage body fat was evaluated by means of electrical impedance analysis instead of dual‐energy X‐ray absorptiometry – which is considered as the gold standard to measure body composition. The training duration under each environmental condition was only 4 weeks. Thus, such a short‐term study does not provide sufficient information regarding reductions in MS risk markers and hs‐CRP levels. Furthermore, HO‐1 was not measured in our study; therefore, it remains unknown exactly what causes decreased hs‐CRP. To validate our findings and to confirm whether hypoxic training reduces the levels of MS risk markers and/or reduces or prevents arteriosclerosis, further studies with a larger number of participants and a long‐term study design are warranted.

In conclusion, we found a significant decrease in PWV, hs‐CRP, waist circumference, and PFT values in our hypoxic training group, suggesting that regular short‐term hypoxic training may more effectively reduce arterial stiffness, inflammation, and central obesity than training performed at a relatively similar exercise intensity under normoxic conditions.

## Conflict of Interest

None declared.
